# Disentangling depression in women with diabetes: evidence for measure-dependent associations with interleukin-4 and common inflammatory biomarkers

**DOI:** 10.3389/fpsyt.2026.1706953

**Published:** 2026-03-20

**Authors:** Nicole Beaulieu Perez, Paula Gordillo Sierra, Jackie Finik, Jason Fletcher, David B. Hanna, Anjali Sharma, Kathryn Anastos, Leah H. Rubin, Gail D’Eramo Melkus, Bradley Aouizerat

**Affiliations:** 1 Rory Meyers College of Nursing, New York University, New York, NY, United States; 2 Departments of Epidemiology & Population Health, Albert Einstein College of Medicine and Montefiore Medical Center, Bronx, NY, United States; 3 Departments of Medicine, Albert Einstein College of Medicine and Montefiore Medical Center, Bronx, NY, United States; 4 Departments of Neurology, Psychiatry and Behavioral Sciences, and Molecular and Cellular Pathobiology, Johns Hopkins School of Medicine, Baltimore, MD, United States; 5 Department of Epidemiology, Johns Hopkins University, Bloomberg School of Public Health, Baltimore, MD, United States; 6 Department of Oral and Maxillofacial Surgery, College of Dentistry, New York University, New York, NY, United States

**Keywords:** affective disorders, depression, depressive symptoms, HIV, inflammation, metabolic health, type 2 diabetes, women

## Abstract

**Background:**

Women living with type 2 diabetes (T2D) face elevated risks for depression and its consequences, including early mortality, yet depression is underrecognized in this population. Depression is a heterogeneous phenotype lacking objective diagnostic biomarkers, with symptoms spanning multiple inconsistently assessed dimensions across various measures. Converging evidence implicates inflammation in depression and links depression with T2D. Here, we explored associations between inflammation and various measures and dimensions of depressive symptoms.

**Methods:**

This cross-sectional pilot study enrolled 38 women with T2D from the MACS/WIHS Combined Cohort Study (MWCCS) Bronx site from 2022-2023. Serum inflammatory biomarkers (hsCRP, INF-*γ*, IL-1β, IL-1RA, IL-2, IL-4, IL-6, IL-8, IL-10, TNF-α) were measured and analyzed via Luminex. We performed Spearman correlation analysis using log-transformed biomarker levels and the multidimensional Center for Epidemiological Studies Depression scale (CES-D), alongside unidimensional Patient-Reported Outcomes Measurement Information System (PROMIS) measures for depression, fatigue, sleep, and anxiety, in this exploratory analysis.

**Results:**

Participants were on average 61.4 (*SD* 4.8) years old, 71% Black, and 32% Hispanic. While all women had T2D, 82% were also living with HIV. Mean BMI of 34.7 kg/m^2^ (*SD* 7.4) was high, but HbA1c of 6.5% (*SD* 1.3%) indicated fairly adequate glycemic control. Among participants with HIV, 94% were taking antiretroviral therapy. Mean high sensitivity C-reactive protein (hsCRP) of 4.1 mg/L (*SD* 3.7) signaled moderate inflammation in this population. IL-4 demonstrated significant negative associations with PROMIS-Depression (*r_s=_* 0.35; 95% CI -0.61, -0.03; *p* = 0.034) and PROMIS-Anxiety scores (*r_s_* = -0.37; 95% CI -0.62, -0.05; *p* = 0.025), but associations with CES-D were not significant. hsCRP and IL-6 were positively correlated with CES-D and negatively correlated with PROMIS-Depression, although these associations did not reach statistical significance. PROMIS-Sleep was moderately associated with IL-8 (*r_s_* = 0.39; 95% CI 0.06, 0.64; *p* = 0.021).

**Conclusions:**

While preliminary, our findings suggest that associations between inflammatory biomarkers and depression may not be consistent across all depressive measures or symptom dimensions. Although larger samples with repeated measures are needed, findings from this exploratory study suggest that including inflammatory measures beyond hsCRP and IL-6, together with tools that capture distinct depressive symptom dimensions, may help to inform future precision mental health research.

## Introduction

Women living with type 2 diabetes (T2D) face elevated risks for depression and its consequences. Depression affects over one-third of women with T2D, accelerates diabetes complications, impairs cognitive, physical, and social functioning, and dramatically increases mortality risk (1, 2). The co-occurrence of depressive symptoms in T2D jeopardizes health and life span by hindering self-care and through several other biopsychosocial mechanisms (3, 4). Despite its significant impact, depression is routinely underrecognized, especially among understudied populations such as Black women and individuals with chronic illnesses, whose symptoms may go unnoticed, disregarded, or overlap with symptoms of cardiometabolic conditions (e.g., fatigue, appetite changes, sleep disturbance) (1, 5, 6). The risk of missed or misattributed symptoms is further compounded by depression heterogeneity and a lack of diagnostic biomarkers (7). Moreover, even when depression is recognized and treated, metabolic balance is not fully restored, suggesting complex underlying mechanisms beyond behavior that may be at play (8, 9).

### Inflammation as a mechanism of depression and link to diabetes co-occurrence

Growing evidence suggests that inflammation may be a key link between depression and T2D. Inflammation can alter the production of neurotransmitters implicated in depression by shifting tryptophan, a precursor to serotonin, toward the production of compounds like kynurenine, which are linked to both mood changes and metabolic problems (10). Inflammatory biomarkers, including high-sensitivity C-reactive protein (hsCRP), interleukin (IL) -6, and tumor necrosis factor alpha (TNF-α), have been frequently linked with both T2D and depression (11–13). While these markers are the most widely studied, they capture only part of the picture. Other less-studied inflammatory signals, such as IL-1β, IL-1 receptor antagonist (IL-1RA), monocyte chemoattractant protein-1 (MCP-1), and interferon-γ (IFN−γ), have been connected to depressive symptoms in people with T2D, even when controlling for factors like weight, sex, and other health conditions (14).

Assessing inflammation may be especially relevant for individuals who show depressive symptoms alongside metabolic issues like high body mass index (BMI), central fat accumulation, or elevated triglycerides, features that often coexist with T2D (15). Inflammation also contributes directly to diabetes by damaging insulin-producing cells and worsening insulin resistance (16). These processes may be mutually compounding in people with HIV (PWH), who face ongoing immune system activation even when the virus is well-controlled (17). However, intersecting depression and inflammatory pathways among individuals with multiple chronic conditions have yet to be robustly explored.

### Lumping heterogeneous symptom-based phenotypes hinders biomarker discovery

Depression is a highly heterogeneous condition, marked by a wide range of symptom profiles that often fluctuate over time and do not have a clear biological categorization (7, 18, 19). Individuals diagnosed with major depressive disorder (MDD) may share the same diagnostic label yet present with entirely distinct constellations of symptoms, ranging from anhedonia and disrupted sleep to cognitive slowing, changes in appetite, fatigue, and anxious features (20). This variability complicates diagnosis for research and practice, particularly in the absence of validated biological markers. Despite decades of study, depression continues to be defined and diagnosed through self-reported symptoms. As illustrated above, the clinical label has been criticized as overly broad, which hinders clinical precision and may obscure the identification of meaningful biological subtypes (18, 21). One such emerging subtype is characterized by immunometabolic dysregulation, where inflammation, metabolic dysfunction, and specific mood symptoms converge (e.g., anhedonia, fatigue, psychomotor retardation, hypersomnia) (7). This phenotype may help explain inconsistent findings in inflammation-related depression research (which aggregates diverse symptom presentations into a single classification) and offers a promising avenue for more targeted, biologically informed interventions. Given the health and economic burdens of depression, including subclinical forms that do not meet diagnostic thresholds but still impair function (22, 23), there is a critical need to move beyond categorical diagnoses toward more precise characterization, particularly in populations with chronic disease.

### Variation in depressive symptom measurement scales

**Figure 1:**
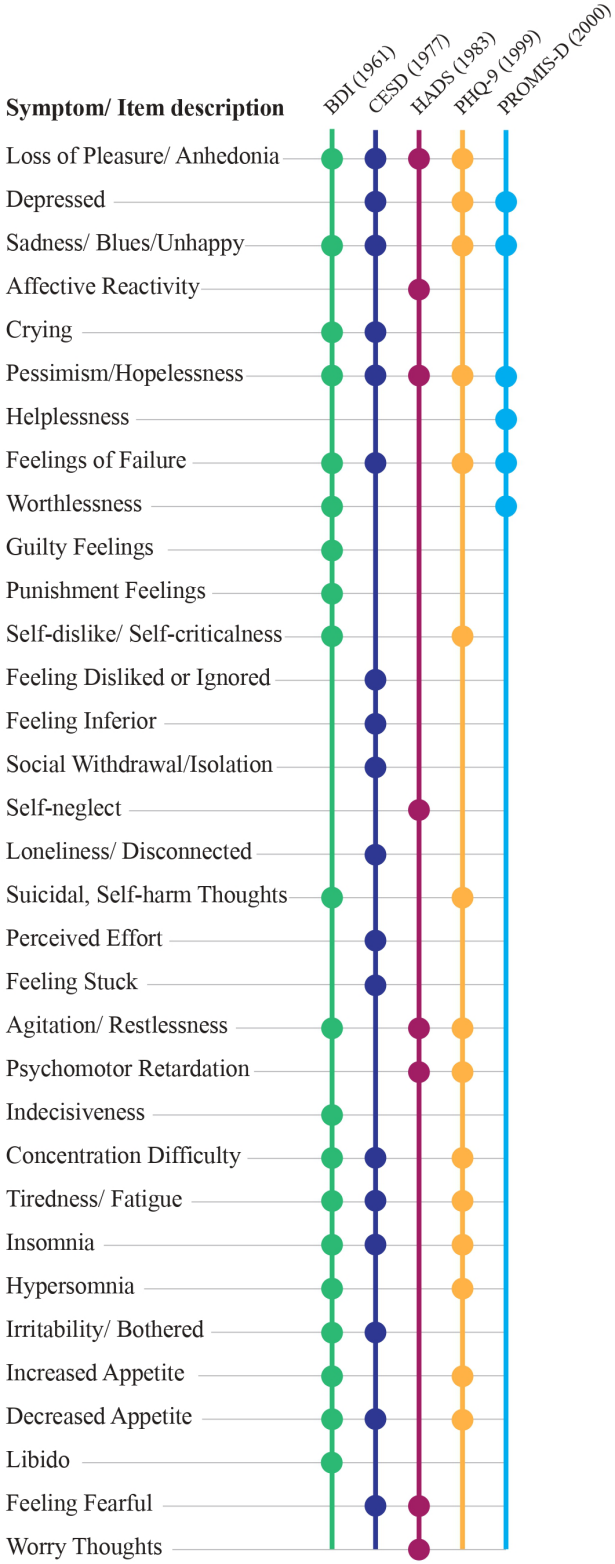
Symptom coverage variance across commonly used depression scales. BDI, Beck Depression Inventory II; CESD, Center for Epidemiological Studies Depression; HADS, Hospital Anxiety and Depression Scale; PHQ-9, Patient Health Questionnaire; PROMIS-D, Patient Reported Outcome Measures Information System- Depression.

Further complicating the issue of depression heterogeneity is the inconsistency and non-equivalence of measurement tools that assess depression severity across divergent symptom dimensions and rarely account for the full range of depressive experiences. As illustrated in **Figure 1**, symptom coverage varies substantially across commonly used scales, with some emphasizing cognitive symptoms (e.g., the Beck Depression Inventory, BDI) others affective or somatic domains [e.g., the Center for Epidemiologic Studies Depression Scale (CES-D) and the Patient Health Questionnaire-9 (PHQ-9)], and still others narrowly reflecting constructs such as anhedonia (e.g., the Hospital Anxiety and Depression Scale) (24). The common practice of generating summary scores from these tools assumes unidimensionality, a premise rarely tested or upheld in older instruments, raising concerns about their validity (21, 25). The Patient-Reported Outcomes Measurement Information System (PROMIS) Depression scale has been identified as a psychometrically rigorous alternative, demonstrating strong structural and content validity and reliability across populations compared to legacy measures like the CES-D and PHQ-9 (26, 27). However, its focus on depressed mood and anhedonia may overlook other symptom dimensions relevant to a full diagnosis of MDD, such as fatigue, sleep disturbance, or cognitive function. Fortunately, PROMIS offers parallel unidimensional scales for these symptoms, enabling researchers to assess them in tandem to yield greater precision.

### Research gaps and study aims

Few studies have examined the granularity in the presentation of depression among individuals with T2D, with many continuing to treat depression as a single, undifferentiated construct. This tendency to collapse diverse symptom profiles into a single diagnosis may obscure biologically meaningful associations and limit the potential for discovery. Addressing this limitation requires expanding the range of inflammatory markers under investigation and moving beyond global depression scores to analyze symptom-level dimensions. Equally important is the comparison of depression measures themselves, as variability in symptom coverage and psychometric rigor may influence both the detection and interpretation of associations. Advancing the field will depend on integrating biologically informed approaches with more refined, multidimensional assessments of depression. To address these gaps, the present study aimed to estimate the effect sizes and assess the consistency of associations between inflammatory biomarkers and multiple measures of depressive symptoms in women with T2D. By exploring these connections across various instruments and a broad inflammatory panel, this work can inform future research and clinical strategies grounded in both biological relevance and measurement precision.

## Methods

The current observational, cross-sectional pilot study aimed to generate effect size estimates needed to inform a larger, fully-powered study to examine relationships between inflammation and depression among women with immunometabolic dysregulation. Methods for the parent cohort and this substudy have been previously described (28, 29). Briefly, we leveraged and collected new data from women enrolled in the Bronx site of the longitudinal Multicenter AIDS Cohort Study (MACS)/Women’s Interagency HIV Study (WIHS) Combined Cohort Study (MWCCS). The MWCCS includes women with HIV and demographically and behaviorally similar women without HIV and collects self-report, laboratory, and physical exam data from participants annually.

### Sample and setting

MWCCS Bronx site participants were enrolled in the current substudy between October 2022 and March 2023. All participants provided written informed consent. Women were eligible for our substudy if they had a diagnosis of T2D (consistent with prior MWCCS studies: fasting glucose ≥126 mg/dL, HbA1C ≥6.5%, or self-reported diagnosis and diabetes medication) and were willing to provide a blood sample (30). Exclusions included pregnancy, inflammatory conditions (e.g., inflammatory bowel disease), and receipt of cancer treatment, as these factors would affect biomarkers and limit study validity.

### Survey measures

To minimize participant burden and expand our analysis, in addition to the biospecimens and survey measures collected for this study, data collected by the MWCCS concurrently (i.e., the most recent visit within 3 months) with substudy data collection were leveraged. Previously collected data included sociodemographic (age, self-reported race and ethnicity, income, education, marital status), clinical indices (BMI, HbA1c, menopausal status, HIV serostatus, CD4 count), health behaviors (smoking, number of alcoholic drinks/week), and depressive symptoms (CES-D) (29).

#### Depressive symptoms

The CES-D is a self-reported, 20-item measure that has long been considered a valid and reliable measure for assessing depressive symptomatology in the general population (31). In the original development population, the CES-D demonstrated a four-factor structure (negative affect, lack of positive affect, somatic, and interpersonal symptoms) (31). However, concerns regarding variability of the CES-D’s factor structure across populations, combined with the problematic practice of creating summary scores (and equal weighting of symptom dimensions) for multidimensional scales, should be noted (21, 32, 33). These concerns challenge the continued use of ‘legacy’ measures, whose development predates the current depression science and psychometric advancements (e.g., item response theory [IRT]).

In contrast, the depression scale created by the PROMIS was born out of an NIH initiative to improve the quality of self-reported outcome measures. Developed using IRT methodology, it is designed to be precise and efficient for patients with various chronic conditions (34). We used the 6-item PROMIS Depression Short Form (PROMIS-Depression), which is unidimensional, has strong reliability and validity, and is consistent with the original computer adaptive testing (CAT) version focused on negative mood, affect, and views of the self (26, 34, 35). Importantly, rather than raw summary scores, the PROMIS measurements are designed to generate standardized T-scores to enhance interpretation and comparison across populations (36).

#### Associated psychological and somatic symptoms

We collected additional PROMIS measures of symptom dimensions associated with depression using 6-item short forms. Specifically, the PROMIS-Anxiety, PROMIS-Fatigue, and PROMIS-Sleep disturbance scales were used as they, like the PROMIS-Depression scale, are highly reliable common data elements based on IRT methods and validated across socio-demographically diverse adult populations with chronic conditions (26, 37). Notably, fatigue, sleep disturbance, and anxiety are part of the DSM-5 essential and specifier criteria for MDD, and represent symptom dimensions also assessed by the CES-D items (20). **Supplementary Table 1** shows how the PROMIS measures provide coverage across similar dimensions assessed by the CES-D, but with the additional rigor and unidimensionality. Measuring individual dimensions using PROMIS measures rather than total scores that aggregate these (e.g., CES-D) allowed us to examine the nuances of inflammation-symptom associations that may be relevant for some but not all presentations of depression.

### Laboratory methods

Blood samples were collected by venipuncture, and Luminex was used to measure serum levels of hsCRP, INF- γ,IL-1RA, IL-1β, IL-4, IL-6, IL-8, IL-10, TNF-α. After collection, blood received standard processing and storage at −80 °C. All samples were collected in the early afternoon to control for diurnal variability. To reduce the potential for batch effects, Luminex analysis using BD™ Cytometric Bead Array was performed after all samples were collected. All samples were run in triplicate on 96-well plates. We used the following criteria to determine if inflammatory analytes, followed by individual cases, should be dropped due to low quality. If the mean fluorescence intensity (MFI) <15,000 or the MFI coefficient of variation (CV) was >20% for more than 20% of values per analyte, that inflammatory measure was dropped. Next, if more than 20% of the inflammatory analytes were suspicious by the above standard criteria for any individual case, that case was excluded. Any remaining values were dropped for violation of quality standards, as noted above.

### Statistical analysis

Descriptive statistics were performed to generate sample characteristics. For greater interpretability, concentration values of inflammatory analytes were used for descriptive characterization. However, to maximize statistical power, MFI values were used in our inferential analysis as the concentration values calculated using a highly conservative standard curve, which showed a moderate level of missingness. Batch effects between plates were assessed using the Mann-Whitney U test, comparing the distributions of each analyte and outcomes of interest across plates.

As inflammatory biomarkers are known to be heavily right-skewed, these MFI values were log-transformed before analysis. This transformation improved but did not completely normalize the analyte distributions; therefore, we used Spearman correlation for non-parametric data to generate correlation coefficients as effect size estimates. We set significance at p<0.05, but our focus is on effect size estimation rather than hypothesis testing, as our sample was not adequately powered for the latter or for the inclusion of covariates. Thus, our reporting of this exploratory analysis focuses on correlation coefficients and confidence intervals while filling a gap in the literature upon which future studies can be built. Given the exploratory design and limited statistical power, effect sizes are emphasized to characterize the magnitude and direction of associations rather than reliance on statistical significance alone. All analyses were conducted in R version 4.4.1.

## Results

Women included in the final analysis (*n* = 38) were on average 61.4 (*SD* 4.8) years old. Most participants identified as Black or African American (71%), and many reported Hispanic ethnicity (31%). Most participants reported completing high school and/or some college (42%), being unemployed, disabled, or retired (92%), not currently partnered (89%), and having a household income of less than ≤$18,000 per year (89%). Mean depression scores, demonstrated by both CES-D 12.49 (*SD* 10.39) and PROMIS-Depression 53.57 (*SD* 9.51), are interpreted as mild to moderate.

Approximately three-quarters of this sample were women with HIV, in whom, the average CD4 was 854 (*SD* 433) cells/mm^3^, and almost all were taking antiretroviral therapy (93%) with high self-reported adherence rates. All participants in this sample were postmenopausal as defined by the Stages of Reproductive Aging Workshop (STRAW)+10 criteria (38). The mean BMI of 34.7 (*SD* 7.4) kg/m^²^ was high, but the mean HbA1c of 6.52 (*SD* 1.32) percent indicated fairly adequate glycemic control. Alcohol consumption was on average 2 (*SD* 5.3) drinks per week, and 21% reported current smoking. The average hsCRP level of 4.1 (*SD* 3.7) mg/L signaled moderate inflammatory elevation. Standard ranges for the other inflammatory measures in this study have not been established. Additional sample characteristics are displayed in **Tables 1** and **2**.

**Table 1:** Sample descriptive characteristics (N = 38).

Characteristic	*M* (*SD*) or n (%)
Sociodemographic	
Age (years)	61.4 (4.8)
Race	
African American or Black	27 (71.1%)
White	1 (2.6%)
Other*(including more than 1 race)	10 (26.3%)
Hispanic or Latino ethnicity	12 (31.6%)
Education	
Less than high school	12 (31.6%)
High school/some college	16 (42.1%)
College graduate or higher	10 (26.3%)
Employment	
Yes	3 (7.9%)
No (Unemployed, retired, disabled, etc.)	35 (92.1%)
Marital status	
Never Married, Separated, Divorced, Widowed	34 (89.5%)
Married or living with partner	4 (10.5%)
Household income ≤$18,000/year	33 (89.2%)
Psychological symptoms ‡	
CES-Depression summary score	12.4 (10.3)
PROMIS-Depression t-score	53.5 (9.5)
PROMIS-Anxiety t-score	54.7 (10.5)
PROMIS-Fatigue t-score	53.2(12.3)
PROMIS-Sleep disturbance t-score	53.8 (9.5)
Clinical Indices	
Body mass index (kg/m²)	34.6 (7.4)
Hemoglobin A1c (%)	6.5 (1.3)
Post-menopausal	38 (100%)
HIV serostatus	
Seropositive	31 (81.5%)
Seronegative	7 (18.4%)
CD4 T-cell count (cells/mm^3^) †	853.7 (432.7)
Undetectable viral load †	31 (100%)
Health Behaviors	
Taking antiretroviral therapy (ART) †	29 (93.5)
ART adherence 100% of the time †	28 (90.3)
Currently smoking	8 (21.1%)
Alcohol drinks/week	2 (5.3)

Values are presented as n (%) for categorical variables and mean (SD) for continuous variables. *”Other” response with free text option to self-identify; **‡** CESD-D ranges from 0-60, ≥ 16 indicating possible depression, PROMIS tools range 0–100 with higher scores indicating worse symptoms and no established cut-offs; †HIV seropositive only.

**Table 2:** Sample inflammatory characteristics (N = 38).

Inflammatory biomarkers	*M* (*SD*)
Inflammatory biomarkers	
hsCRP (mg/L)	4.08 (3.7)
INF-g (pg/mL)	5.22 (19.85)
IL-1b (pg/mL)	9.42 (7.20)
IL-1ra (pg/mL)	2.62 (2.53)
IL-2 (pg/mL)	2.42 (1.16)
IL-4 (pg/mL)	1.54 (2.34)
IL-6 (pg/mL)	3.18 (3.48)
IL-8 (pg/mL)	1.32 (1.42)
IL-10 (pg/mL)	1.78 (1.46)
*TNF*-a (pg/mL)	12.26 (7.41)

### Preliminary evidence of associations between interleukin-4 and depression and anxiety symptoms

**Figure 2:**
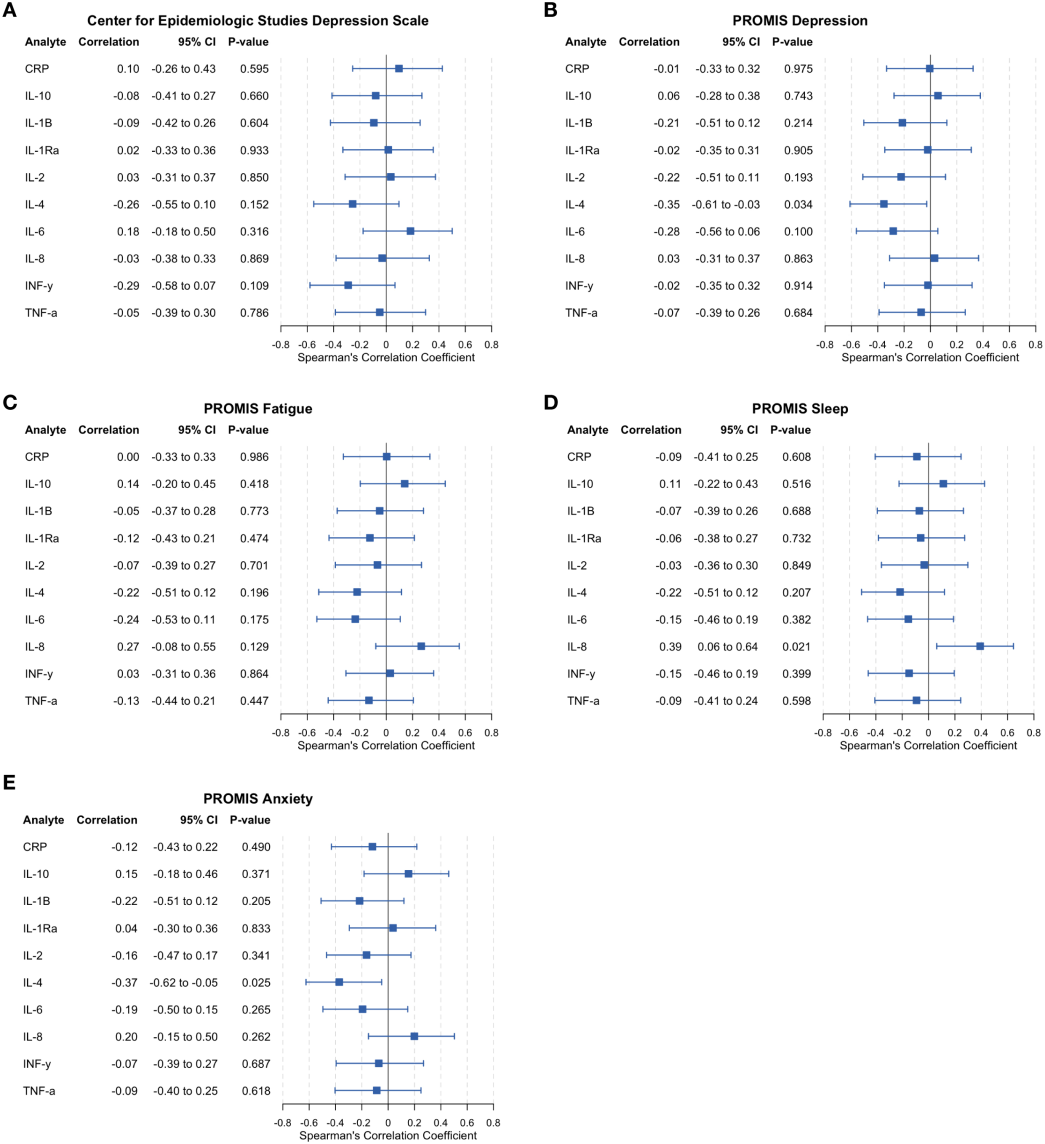
Correlations between inflammatory biomarkers and symptom measures. Forest plots show correlation between inflammatory biomarkers and patient-reported outcomes; horizontal lines indicate 95% confidence intervals. **(A)** Center for Epidemiologic Studies Depression Scale (CES-D); **(B)** PROMIS Depression; **(C)** PROMIS Fatigue; **(D)** PROMIS Sleep Disturbance; **(E)** PROMIS Anxiety. Red indicates statistically significant.

Spearman correlations demonstrated statistically significant negative associations between IL-4 and PROMIS-Depression (*r_s_* = -0.35; 95% CI -0.61, -0.03; *p* = 0.034) and PROMIS-Anxiety scores (*r_s_* = -0.37; 95% CI -0.62, -0.05; *p* = 0.025), representing moderate effect sizes (**Figures 2B, E**). Other inflammatory markers demonstrated small to moderate negative correlations with PROMIS-Depression, including IL-1β (*r_s_* = -0.21; 95% CI -0.51, 0.12; *p* = 0.214), IL-2 (*r_s_* = -0.22; 95% CI -0.51, 0.11; *p* = 0.193), and IL-6 (*r_s_* = -0.28; 95% CI -0.56, 0.06; *p* = 0.1). PROMIS-Anxiety scores were also negatively associated with IL-1β (*r_s_* = -0.22; 95% CI -0.51, 0.12; *p* = 0.205) and IL-6 (*r_s_* = -0.19; 95% CI -0.05, 0.15; *p* = 0.265), although these did not reach statistical significance. Remaining inflammatory biomarkers, including hsCRP, IL-1RA, IL-8, IL-10, IFN-γ, and TNF-α were not associated with PROMIS-Depression or PROMIS-Anxiety scores.

### C-reactive protein and interleukin-6 across depression measurement scales

No statistically significant relationships were detected between inflammatory biomarkers and CES-D scores; however, hsCRP (*r_s_* = 0.10; 95% CI -0.26,0.43; *p* = 0.595) and IL-6 (*r_s_* = 0.18; 95% CI -0.18, 0.50; *p* = 0.316) demonstrated positive associations with CES-D at small effect sizes. In line with the direction observed from PROMIS-Depression scores, IL-4 was moderately negatively associated with CES-D (*r_s_* = -0.26, 95% CI -0.55, 0.10; *p* = 0.152). In addition, INF-y demonstrated moderate negative associations (*r_s_* = -0.29; 95% CI -0.58, 0.07; *p* = 0.109) with CES-D scores, but neither reached statistical significance. As shown in **Figures 2A, B**, hsCRP was positively associated with CES-D scores. In contrast, there was no correlation between hsCRP and PROMIS-Depression (*r_s_* = -0.01; 95% CI -0.33, 0.32; *p* = 0.975), and the direction of the association of IL-6 reversed between the two depression measures (effect size noted above). These results must be interpreted cautiously as exploratory and hypothesis-generating, given our underpowered pilot sample, our inability to control for clinical heterogeneity, and the risks of false positives in light of multiple comparisons.

### Interleukin-8 positively associated with sleep and fatigue

Higher PROMIS-Sleep disturbance was moderately associated with higher levels of IL-8 (*r_s_* = 0.39; 95% CI 0.06, 0.64; *p* = 0.021). Similarly, higher levels of fatigue were associated with IL-8 (*r_s_* = 0.27; 95% CI -0.08, 0.55; *p* = 0.129). Small to moderate negative associations between sleep and fatigue were also observed for IL-4, and lower levels of IL-6 were associated with higher levels of fatigue (**Figures 2C, D**). Small and non-significant effects were observed for all other biomarkers correlated with sleep disturbance and fatigue.

## Discussion

A growing literature examines the relevance of inflammation in depression and depressive symptoms, but associations between varied depression measures and symptom dimensions with inflammation have yet to be explored. The present study estimated the effect sizes of associations of a legacy depression measure (CES-D) and newer, unidimensional, PROMIS measures with several inflammatory biomarkers among a pilot sample of women with T2D from a HIV cohort study. In our sample of women, predominantly living with HIV with mild to moderate depressive symptoms, we observed moderate associations of IL-4 with CES-D and PROMIS measures of depression and anxiety. However, contradictory associations were observed for CRP and IL-6 when CES-D was juxtaposed with the PROMIS-Depression measure. To our knowledge, this is the first study to explore associations of inflammation across multiple measures of depression and symptom dimensions. However, this study was not powered for confirmatory analyses; the findings should be interpreted as exploratory.

Our findings that higher levels of depressed mood and anxiety may associate with lower levels of IL-4 align with prior pre-clinical and clinical studies. IL-4 is a pleiotropic cytokine, as it exhibits a range of effects, including context-dependent pro- and anti-inflammatory activities, although it has previously been labeled anti-inflammatory (39, p. 4; 40). Although our findings are but preliminary signals, other studies suggest a therapeutic potential of IL-4 in CNS disorders, including experimental stroke, spinal cord injury, and animal models of multiple sclerosis and depression (39, 41). *In vitro* models aimed to elucidate molecular mechanisms of IL-4 treatment suggest that glial cells secrete neurotropic factors (including brain-derived neurotrophic factor [BDNF]) in response to IL-4. (39). Human trials of IL-4 have yet to be conducted, but low IL-4 has been observed with depression across clinical populations (40, 42).

Less is known about IL-4 and anxiety, and our findings may be related to the strong link between depression and anxiety symptoms themselves. Evidence concerning IL-4 and anxiety is mostly limited to animal studies (43), but some small studies have reported altered IL-4 levels in individuals with anxiety (44). However, the inconsistent findings underscore an important gap.

The positive associations between hsCRP and IL-6 with CES-D scores are in line with an established literature, including meta-analyses, linking inflammation and depression (11, 12), but the inconsistency of the direction of these associations across depression measures (i.e., CES-D versus PROMIS) raises questions about the quality and consistency across these tools. The absence of statistically significant associations between these markers and CES-D scores could be attributable to limited statistical power given our small sample size, but may also reflect the clinical and demographic characteristics of our sample. Specifically, our study was comprised predominantly of Black, postmenopausal women. Previous studies examining the influence of sex and race have suggested links between inflammation and depression are strongest among men and White women (45, 46), yet these studies do not report on the reproductive status despite largely mid-life samples. A growing body of literature suggests that fluctuations and decreases in estrogen and other ovarian hormones may be linked to inflammation and depressive symptoms during the perimenopausal period, underscoring the importance of considering this biological variance across samples (47, 48). In addition, our study included women with T2D and HIV, increasing the risk of confounding and limiting comparability as the majority of similar studies exclude participants with these comorbid medical conditions, but not necessarily acute infection (e.g. upper respiratory infection), which was a strength of the present study (12). Furthermore, prior studies have been conducted among predominantly mid-life, medically healthy men and women using multidimensional legacy measures of depression (e.g, CES-D, BDI), inconsistent with modern IRT method standards for patient-reported outcomes (11, 12, 21, 49).

IL-8 is a proinflammatory cytokine involved in immune signaling and has been increasingly recognized for its relevance across a variety of health conditions. Its association with sleep and fatigue has been noted in both clinical and preclinical studies, and our observed association between IL-8 and sleep disturbance is consistent with this growing evidence (50, 51). Beyond sleep-related outcomes, altered IL-8 has been observed in mood disorders, particularly inflammation-linked depression (52), as well as in infectious, neurological, and inflammatory diseases. Elevated IL-8 levels have been observed in conditions such as COVID-19 (53), colorectal cancer (54), Parkinson’s disease (55), pain and fatigue in lung cancer patients (56), often correlating with symptom severity or disease progression.

### Advancing science by considering transdiagnostic symptom dimensions and grounding inquiry in theory

A unique contribution of this study was our attention to transdiagnostic symptoms using the PROMIS measures to explore inflammation-associated dimensions and set the stage for research powered to decipher subphenotypes. Among otherwise healthy samples with depression, an inflammation-linked subphenotype of depression has been characterized by anhedonia, elevated CRP, altered glucose and tyrosine (a dopamine precursor) metabolism (57), but the prevalence of this phenotype among populations with chronic illness is unknown. Our tandem exploration of symptom dimensions is also consistent with the Research Domain Criteria (RDoC) framework proposed by NIMH (58), and we observed variance across dimensions of depressive symptoms, albeit on a preliminary basis. This approach is congruent with recognition of depression as a multidimensional construct spanning several domains and enables testing of alternative theories of depression. Although the DSM is explicitly atheoretical, the implication of many legacy measurement tools is that depression symptoms are a manifestation of an underlying disease consistent with the common cause theory of depression, in which all symptoms spring from a central source (21). Employment of unidimensional tools (e.g., PROMIS measures) enable testing of alternative theories of depression such as *network theory*, which posits that symptoms (also called features or nodes) interact with each other and factors in the external field (e.g., inflammation, stress) forming feedback loops that can result in the sustained and overlapping disordered states often seen in clinical care (21, 59).

### Strengths and limitations

This study offers important preliminary insights into the associations between inflammation and depressive symptoms in women with T2D; however, results must be interpreted cautiously due to several methodological constraints. An important limitation is the potential for confounding, specifically by HIV status. The small sample size and unequal distribution of HIV (81.5%) in our sample prevented robust sensitivity analyses, constraining our ability to determine whether observed inflammatory patterns are specific to depressive symptom dimensions or reflect broader HIV-related immune processes. Other relevant factors, including body mass index, smoking, menopausal status, and glycemic control, were described but not controlled, and future studies should consider these important covariates in fully powered analysis. Additionally, although the consistency of IL-8 findings with prior research boosts confidence, the risk of false positives resulting from uncorrected multiple testing cannot be ignored. Although the sample size was small and not designed to address definitive questions about causal links between inflammation and depression, the results provide effect-size estimates that may inform the design of future studies. Relatedly, the cross-sectional design precludes determining temporality and parsing cause from consequence.

This study also included several design strengths. PROMIS assessments and blood draws were completed on the same day, and potential confounders, such as acute infection, were reduced through strict exclusion criteria and scheduling procedures. Although CES-D scores were not collected on the same day, they were drawn from within the study visit window to minimize variability. Inflammatory markers are dynamic, and their variability over time may have introduced measurement error; however, all samples were collected at a consistent time of day, and no associations were found between time since waking and either depressive symptoms or inflammatory levels. Lastly, while the PROMIS-Depression scale used in this study offers strong psychometric qualities, it does not assess cognitive symptoms such as concentration difficulties, which are captured by the CES-D, which may be relevant to gather a comprehensive understanding of depression in this population.

## Implications and conclusions

Our study has several implications for future research, clinical practice, policy, and education. Clinicians, particularly those practicing in primary care settings, need to recognize that depression is a multidimensional syndrome that varies significantly in presentation from one person to the next, as this understanding is critical for timely identification and early intervention. Although efforts to increase measurement-based care in mental health remain prudent and linked to improved outcomes and patient engagement, our observations raise questions about the limitations in many commonly used measures, both in flawed scoring methods that sum across distinct symptom dimensions and the variation in symptoms assessed across many measures (21). A holistic assessment of a patient’s change from baseline across affective, somatic, cognitive, and social symptom dimensions is necessary to inform clinical decisions, including diagnosis and interventions tailored to each patient’s specific symptom profile. Yet this aspirational care standard may be unrealistic in the context of today’s fragmented encounter-based care. Policies that promote and protect systems of such care continuity should be prioritized to advance mental health for all.

More research is needed to understand how inflammation is related to depressive symptoms among women with T2D and HIV. Larger samples with greater variability across depressive symptom severity are needed to extend these findings and examine the role of environmental factors and health behaviors that may contribute to both inflammation and depression in this population, as well as the potential moderating role of HIV and glycemic control. Longitudinal designs that examine women during the progression of T2D or during the menopausal transition would help clarify the direction of relationships. This would be further enhanced by the use of multiple unidimensional symptom scales to explore the potential effects of symptom interaction and test underlying depression theories. Such knowledge would meaningfully advance efforts toward the development of precision prevention and intervention strategies in mental health care.

## Data Availability

The data analyzed in this study is subject to the following licenses/restrictions: Data collected from the MACS/WIHS Combined Cohort Study (MWCCS) Bronx site from 2022-2023. Data from this study are not publicly available. They may be available by emailing the corresponding author upon reasonable request. Requests to access these datasets should be directed to Nicole Beaulieu Perez, nbp273@nyu.edu.
