# Supplementary material for: Disentangling depression in women with diabetes: evidence for measure-dependent associations with interleukin-4 and common inflammatory biomarkers
Source: Front Psychiatry. 2026 Mar 20;17:1706953. doi: 10.3389/fpsyt.2026.1706953 (PMC13047129; doi:10.3389/fpsyt.2026.1706953)
Supplement: Supplementary file 1 [file Table1.docx]

Supplemental Table 1.
*Comparison of Symptoms Queried Across CES-D and PROMIS Depression, Anxiety, Sleep, and Fatigue*

| Symptom/Item description | Center for Epidemiological Studies-Depression | | PROMIS-Depression SF6a | PROMIS-Anxiety SF6a | PROMIS-Fatigue SF6a | PROMIS-Sleep SF6a |  |
| --- | --- | --- | --- | --- | --- | --- | --- |
| Loss of Pleasure/ Anhedonia | | 16 |  |  |  |  |  |
| Depressed | | | 6 | 3 |  |  |  |
| Sadness | | 18 | 6 |  |  |  |  |
| Feeling Blue | | 3 |  |  |  |  |  |
| Unhappy | | 12 | 6 |  |  |  |  |
| Crying | | 17 |  |  |  |  |  |
| Hopelessness | | 8 | 4 |  |  |  |  |
| Helplessness | |  | 2 |  |  |  |  |
| Feelings of Failure | | 9 | 5 |  |  |  |  |
| Feeling Disliked or Ignored | | 19 |  |  |  |  |  |
| Feeling Inferior | | 4 |  |  |  |  |  |
| Worthlessness | |  | **1** |  |  |  |  |
| Talk less | | 13 |  |  |  |  |  |
| Loneliness | | 14 |  |  |  |  |  |
| Perceived Effort | | 7 |  |  |  |  |  |
| Feeling Stuck | | 20 |  |  | 2 |  |  |
| Loss of Energy | |  |  |  | 2 |  |  |
| Fatigue | |  |  |  | 1, 3, 5 |  |  |
| Reduced function | |  |  |  | 6 |  |  |
| Reduced coping | |  |  |  | 4 |  |  |
| Insomnia | |  |  |  |  | 3, 6 |  |
| Restless Sleep | | 11 |  |  |  | 5 |  |
| Sleep Quality | |  |  |  |  | 1, 2 |  |
| Difficulties With Sleep | |  |  |  |  | 4 |  |
| Refreshing Sleep | |  |  |  |  | 2 |  |
| Hours of Sleep | |  |  |  |  | 7 |  |
| Irritability/Unusually Bothered | | 1 |  |  |  |  |  |
| Decreased Appetite | | 2 |  |  |  |  |  |
| Concentration Difficulty | | 5 |  | 3 |  |  |  |
| Feeling Fearful | | 10 |  | 1 |  |  |  |
| Need Help for Anxiety | |  |  | 2 |  |  |  |
| Overwhelming worries | |  |  | 4 |  |  |  |
| Nervousness/Uneasy | |  |  | 5,6 |  |  |  |

*Note:* Numbers indicate the item number corresponding to each measure.
